# Heat Shock Protein Upregulation Supplemental to Complex mRNA Alterations in Autoimmune Glaucoma

**DOI:** 10.3390/biom12101538

**Published:** 2022-10-21

**Authors:** Sabrina Reinehr, Armin Safaei, Pia Grotegut, Annika Guntermann, Teresa Tsai, Stephan A. Hahn, Steffen Kösters, Carsten Theiss, Katrin Marcus, H. Burkhard Dick, Caroline May, Stephanie C. Joachim

**Affiliations:** 1 Experimental Eye Research Institute, University Eye Hospital, Ruhr-University Bochum, In der Schornau 23-25, 44892 Bochum, Germany; 2 Department Functional Proteomics, Medizinisches Proteom-Center, Ruhr-University Bochum, ProDi E2.227, Gesundheitscampus 4, 44801 Bochum, Germany; 3 Department of Molecular GI Oncology, Faculty of Medicine, Ruhr-University Bochum, 44780 Bochum, Germany; 4 Department of Cytology, Institute of Anatomy, Ruhr-University Bochum, Universitaetsstr. 150, 44801 Bochum, Germany

**Keywords:** Cxcl10, Gdf15, glaucoma, Hba-a1, heat shock protein, immune system, microarray, Wwox

## Abstract

Glaucomatous optic neuropathy is a common cause for blindness. An elevated intraocular pressure is the main risk factor, but also a contribution of the immune system seems likely. In the experimental autoimmune glaucoma model used here, systemic immunization with an optic nerve homogenate antigen (ONA) leads to retinal ganglion cell (RGC) and optic nerve degeneration. We processed retinae for quantitative real-time PCR and immunohistology 28 days after immunization. Furthermore, we performed mRNA profiling in this model for the first time. We detected a significant RGC loss in the ONA retinae. This was accompanied by an upregulation of mRNA expression of genes belonging to the heat shock protein family. Furthermore, mRNA expression levels of the genes of the immune system, such as *C1qa*, *C1qb*, *Il18*, and *Nfkb1*, were upregulated in ONA animals. After laser microdissection, inner retinal layers were used for mRNA microarrays. Nine of these probes were significantly upregulated in ONA animals (*p* < 0.05), including *Hba-a1* and *Cxcl10*, while fifteen probes were significantly downregulated in ONA animals (*p* < 0.05), such as *Gdf15* and *Wwox*. Taken together, these findings provide further insights into the pivotal role of the immune response in glaucomatous optic neuropathy and could help to identify novel diagnostic or therapeutic strategies.

## 1. Introduction

Among adults, one of the most common causes for visual impairment is glaucoma [1,2]. In this disease, a loss of retinal ganglion cells (RGCs) and optic nerve degeneration lead to progressive visual field loss, which, untreated, can end in blindness [3].

Although elevated intraocular pressure (IOP) is a major risk factor, other mechanisms seem also to be responsible for the onset and progression of glaucomatous damage. Around 30–40% of Caucasians develop glaucoma with an IOP in the normal range [4,5]. At the molecular level, it has clearly been shown that oxidative stress [6], ischemia [7,8], or excitotoxicity [9,10] contribute to the neurodegenerative processes. Furthermore, immunological alterations seem to play a crucial role in the pathogenesis of glaucoma [11,12]. For example, several heat shock proteins (HSPs), such as HSP27, HSP60, or αA- and αB-crystallin, were upregulated in patients diagnosed with normal-tension glaucoma [13,14]. Furthermore, an enhanced immunoreactivity against HSP27 and HSP60 antibodies was observed in human donor retinae from patients with and without elevated IOP compared to respective controls [15]. Among other functions, HSPs can stimulate both the innate and the adaptive immune system [16,17]. As part of the innate immune response, activation of the complement system can be observed in glaucoma patients. For instance, the complement factor 3 (C3) is upregulated in the aqueous humor of patients with primary open-angle glaucoma (POAG), particularly in those during disease progression [18,19]. Moreover, complement proteins, including C3 or proteins associated with the lectin pathway, were found in retinae and sera of POAG patients [6,20].

To determine the relation of an altered immune response and glaucomatous optic neuropathy more precisely, the experimental autoimmune glaucoma model (EAG) was established. In this model, systemic immunization with ocular antigens, such as the optic nerve homogenate antigen (ONA) or HSP27, led to optic nerve and RGC degeneration in rats as well as in mice [21,22,23,24]. This was accompanied by an activation of the immune system, particularly, a complement and microglia response [21,22,25].

Interestingly, we were able to make these observations not only on protein, but also on an mRNA level. For example, two weeks after ONA immunization, an upregulation of *C3* as well as of phosphacan (*Ptprz1*), part of the extracellular matrix, could be detected using RT-qPCR [25,26].

To underline our previous findings, DNA microarray analysis was performed. This “top-down” approach enables the investigation of altered gene expression on a primary molecular level. For the first time, primary mRNA alterations that may play a role in the development of glaucomatous optic neuropathy were identified here in a pressure independent EAG model. Overall, our remarkable results may contribute to the development of glaucoma disease markers and novel therapies.

## 2. Materials and Methods

### 2.1. Animals and Immunizations

Animal experiments and animal care procedures adhered to the ARVO Statement for Use of Animals in Research and the animal care committee of North Rhine-Westphalia (approval codes: 84-02.04.2013.A291 and 81-02.04.2018.A071). In this study, six-week-old male Lewis rats (Charles River; Sulzfeld, Germany) were included. All animals had unlimited access to food and water and were kept on a light–dark cycle (12 h:12 h). Health checks and eye exams were performed regularly.

**Figure 1:**
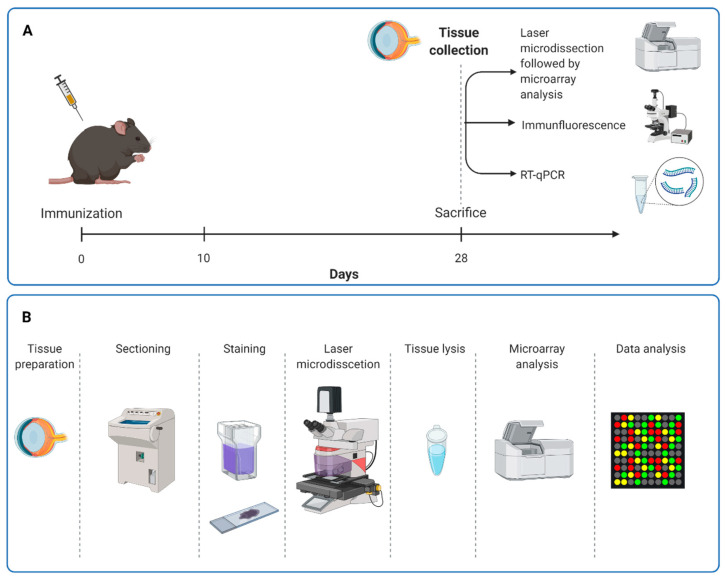
Study design. (**A**) Rats received an intraperitoneal injection with ONA or sodium chloride solution. At 28 days after immunization, the animals were sacrificed, and laser microdissection of the retina followed by microarray analyses were performed. Furthermore, immunohistological staining and RT-qPCR analyses were carried out. (**B**) For microarray analyses, eyes were removed and retinal cross-sections provided. Cresyl violet staining was used to visualize retinal layers, especially neuronal cells. GCL, IPL, and INL were labeled and dissected out by a laser. After dry collection, RNA was isolated for microarray analysis. GCL = ganglion cell layer; IPL = inner plexiform layer; INL = inner nuclear layer.

Systemic immunization of rats was performed as described previously [27] (Figure 1A). Rats received an intraperitoneal injection with 8 mg/mL ONA mixed with incomplete Freund’s adjuvant (500 µL) plus 3 µg pertussis toxin (both Sigma Aldrich, St. Louis, MO, USA) [28]. The animals of the control group were injected with a sodium chloride solution in Freund’s adjuvant and pertussis toxin. Animals were sacrificed 28 days after immunization.

### 2.2. Electroretinography

At 28 days after immunization, electroretinogram (ERG) measurements were performed (*n* = 8 eyes/group). Therefore, rats were dark-adapted the night before the analyses. The function of the retina was monitored using full-field flash electroretinography (HMsERG system; OcuScience, Henderson, NV, USA) [8,28]. Scotopic flash ERGs were recorded at 0.1, 0.3, 1, 3, 10, and 25 cd.s/m^2^. Signals obtained from the corneal surface were amplified, digitized, averaged, and stored using commercial software (ERGView 4.380R; OcuScience) for later analysis. Data were filtered with 50 Hz before evaluating amplitudes of the a- and b-wave and statistical analyses were performed using Statistica (V13; Dell, Tulsa, OK, USA).

### 2.3. Quantitative Real-Time PCR

For RNA preparation, retinae were isolated and transferred to lysis buffer containing 2-mercaptoethanol (Sigma-Aldrich, *n* = 3–4 eyes/group). Total RNA was extracted using the GeneElute Mammalian Total RNA Miniprep Kit according to the manufacturer’s instructions (Sigma-Aldrich) and digested with RNase-free DNase I (Sigma-Aldrich). Quality and quantity of RNA were determined using a NanoDrop ONE (Thermo Fisher Scientific, Waltham, MA, USA). Total RNA (1 µg) was used for reverse transcription using a cDNA synthesis kit (Thermo Fisher Scientific).

Designed oligonucleotides are listed in Table 1. Real-time PCR (PikoReal RT-qPCR Cycler, Thermo Fisher Scientific) analyses were performed using DyNAmo Flash SYBR Green (Thermo Fisher Scientific). Oligonucleotide concentration was optimized to a final concentration of 200 nM and combined with 200 ng of retinal cDNA per well. Two reactions were set up per RNA sample (duplicates) with an end volume of 20 µL per single reaction. Each RT-qPCR was performed in duplicates from each retina (*n* = 3–4/group). The average threshold cycle (Ct) values of the two independent sets were used to calculate the ratios for the target genes. The Ct values of two reference genes (*Actb* and *Ppid*) were considered [28].

### 2.4. Immunohistology of Retinal Sections

After 28 days, eyes were enucleated and fixed for 1 h in 4% paraformaldehyde (*n* = 7 eyes/group). Following a 30% sucrose treatment, they were embedded in Tissue Tek (Thermo Fisher Scientific). Cross-sections (10 µm thickness) were cut with a cryostat and mounted onto Superfrost Plus (both Thermo Fisher Scientific) or Histobond slides (Paul Marienfeld GmbH&Co KG., Lauda-Königshofen, Germany) for further immunohistological analysis.

Specific immunofluorescent antibodies were used to identify the different cell types in the retina (*n* = 7/group). Concisely, retinal sections were blocked with a solution containing 20% donkey serum/10% goat serum, and 0.1–0.2% Triton-X in PBS. Then, 1% bovine serum albumin was added in specific stains. The primary antibodies were incubated overnight at room temperature (Table 2). Incubation with corresponding secondary antibodies was performed for 1 h the next day (Table 2). Nuclear staining with 4′,6-diamidino-2-phenylindole (DAPI) was carried out for better orientation on the slides. Negative controls for all stains were obtained by only applying the secondary antibodies.

### 2.5. Evaluation of Immunohistological Stainings

Images of retinal cross-sections were acquired using an Axio Imager M2 microscope (Zeiss, Oberkochen, Germany). Per cross-sections, four images (two central and two peripheral) were taken. Subsequently, equal areas of each image were cut out (Corel Paint Shop Pro X8, Corel Corporation, Ottawa, ON, Canada). Furthermore, the number of RBPMS^+^, C1q^+^, and NFκB^+^ cells was counted in the ganglion cell layer (GCL) using ImageJ software (National Institute of Health, Bethesda, MD, USA).

The positive staining area of GFAP and HSP25 was measured using an ImageJ macro as described previously [29,30]. Briefly, images were transferred into grayscale (32-bit) and a defined rolling ball radius (GFAP and HSP25: 50 pixels) was subtracted to minimize background interference. Furthermore, a suitable lower threshold was determined for each picture, which was achieved when the grey scale picture corresponded to the original one. At the end, the mean value was calculated, and this number was used for the final analyses (GFAP: 11.11; HSP25: 17.59). The upper threshold was set as the highest number out of all pictures (GFAP and HSP25: 255.00). Between these defined lower and upper thresholds, the percentage of the labeled area was measured.

### 2.6. Sample Preparation for mRNA Microarrays

All operational steps were performed under sterile RNAse free conditions. After enucleation, the eyes were immediately frozen in liquid nitrogen. To obtain retinal cross-sections, the frozen eye was placed on a platelet with the help of NEG-50 Tissue Tek medium (Thermo Fisher Scientific, Waltham, MA, USA). Then, 20 μm thick cross-sections were cut using a cryostat with a fixed knife holder (−24 °C object temperature and −20 °C chamber temperature; Thermo Fisher Scientific) and taken up on 1.0 PET NF membrane slides optimized for laser microdissection (Zeiss) and dried for 2 min by −20 °C. Sections were stored at −80 °C until further use. Subsequently, sections were stained using cresyl violet as described in detail before by Aring et al. 2018 [31]. Briefly, the tissue was dipped in 70% ethanol for 2 min. Then, the tissue was first immersed in a cresyl violet staining solution (1% (*w*/*v*) cresyl violet in 50% ethanol) and then, in 100% ethanol for 30 s (Figure 1B).

The laser microdissection was performed as described before by Plum et al., 2016, with minor changes (*n* = 3 eyes/group) [32]. With the use of the bright field microscope of the laser microdissection device (PALM Micro Beam, P.A.L.M.-System, Zeiss), the retinal layers of interest (GCL, inner plexiform layer (IPL) and inner nuclear layer (INL)) were marked using software supplied by the manufacturer (PALMRobo 4.6 pro, Zeiss). Then, a reaction tube (AdhesiveCap 500 opaque, Zeiss) was applied to the instrument and directly placed above the slide. The retinal regions of interest were cut and catapulted with a laser into the sample cap. Finally, the samples contained about 13,000.00 µm^2^ of retinal tissue. The collected tissue was removed from the lid of the reaction tube with the help of a lysis buffer (RNAqueous Micro Kit, Ambion, now Thermo Fisher Scientific). After placing the 10 µL buffer on the surface of the cap, the reaction tube was closed and turned upside down for 30 min to allow the buffer to detach the cut material from the silicone layer. After the incubation time, the sample was shortly centrifuged down and frozen until further processing. In order to perform microarray analysis, the RNA was isolated using the RNAqueous Micro Kit according to the manufacturer’s instructions and stored at −80 °C until further usage (Figure 1B).

### 2.7. Microarray Analyses

For microarray analyses, we followed the steps as described previously by Puscz et al. [33]. Each RNA sample (100 ng) was hybridized to Agilent whole-genome expression microarrays (Rat GE 4x44K, v3 G2514F, AMADID 028282, Agilent Technologies, Santa Clara, CA, USA). The next steps, including RNA labeling, hybridization, and washing, were carried out according to the instructions of the manufacturer. A DNA microarray scanner (Agilent G2505C) was used to acquire images of the hybridized microarrays. Subsequently, features were extracted using the Agilent Feature Extraction image analysis software (AFE; version A.10.7.3.1) with default protocols and settings. The generated single intensity measure for each feature, referred to as the total gene signal (TGS), was used for further data analyses using the GeneSpring GX software package (Version 14.9.1). AFE–TGS were normalized by the quantile method and data were than filtered on normalized expression values.

Duplicates were carried out for each sample. To identify differentially expressed genes, we included only entities where at least 4 out of the total number of samples had values within the selected cut-off (50th–100th percentile) for subsequent data analysis process. This resulted in 345 differentially expressed genes (GeneSpring GX software package version 14.9.1). At the end, only mRNAs with a *p*-value below *p* < 0.050 were further considered in the microarray analysis (Supplement Table S1).

### 2.8. Statistics

For RT-qPCR analyses, the relative expression values were presented as median ± quartile ± minimum/maximum. Data were assessed by Pairwise Fixed Reallocation Randomization Test© using REST© software (Qiagen, Hilden, Germany) [34]. ERG and immunohistology data are presented as mean±SEM unless otherwise noted. Microarray data are shown as normalized fluorescence intensity. The ONA group was compared to the controls using two-tailed Student’s *t*-test using Statistica. P-values below *p* < 0.05 were considered statistically significant with * *p* < 0.05; ** *p* < 0.01, and *** *p* < 0.001.

## 3. Results

### 3.1. No Functional Alterations after Immunization, but Loss of Retinal Ganglion Cells

**Figure 2:**
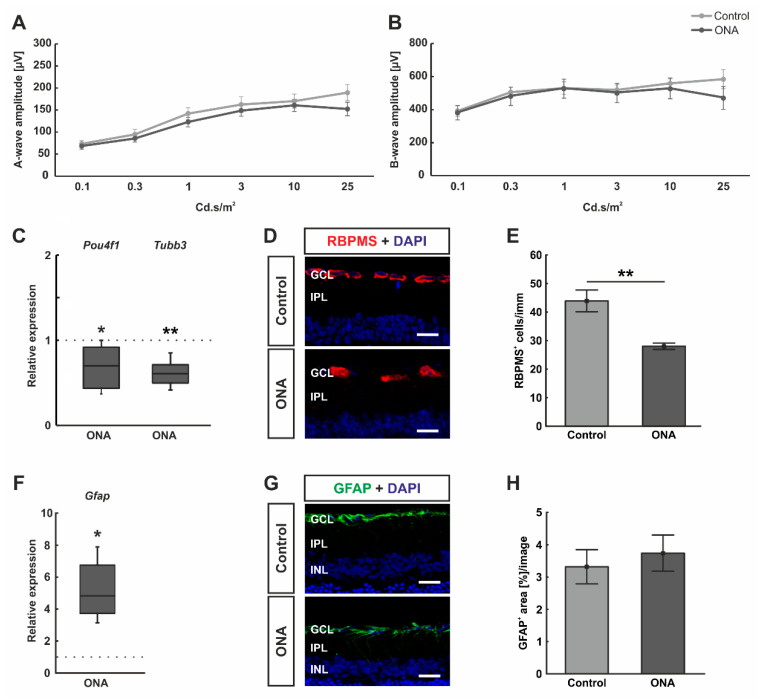
No functional alteration, but loss of retinal ganglion cells after immunization. (**A**) Electrophysiological analyses were performed 28 days after ONA immunization. The response of the photoreceptors is depicted as the a-wave amplitude. At all measured light intensities, the a-wave amplitudes did not differ between ONA and control animals. (**B**) In addition, the b-wave amplitudes, which reflect the response of the inner nuclear cells, were not altered in ONA retinae compared to control ones at all light intensities. (**C**) RT-qPCR analyses revealed a significant downregulation of *Pou4f1* (*p* = 0.036) and *Tubb3* mRNA expression levels (*p* = 0.006) in ONA rats. (**D**) RGCs were labelled against anti-RBPMS (red) and cell nuclei were counterstained with DAPI (blue). (**E**) The number of RGCs were significantly decreased in ONA animals compared to controls (*p* = 0.004). (**F**) *Gfap* mRNA expression levels were significantly upregulated after ONA immunization (*p* = 0.027). (**G**) Retinal cross-sections were stained against anti-GFAP (astrocytes; green) and DAPI (cell nuclei; blue). (**H**) The GFAP^+^ staining area was not altered between ONA and control animals. Scale bars: 20 µm. GCL = ganglion cell layer; IPL = inner plexiform layer; INL = inner nuclear layer. Values for RT-qPCR are median ± quartile ± minimum/maximum and values for ERG and immunofluorescence are mean ± SEM. The dotted lines in (**C**,**F**) represent the relative expression of the control group. * *p* < 0.05, ** *p* < 0.01.

ERG measurements were performed in control as well as ONA animals 28 days after immunization. The results revealed that the a-wave amplitude, reflecting the photoreceptor response, was not altered at any light intensity between both groups (*p* > 0.05; Figure 2A). Furthermore, the amplitudes of the b-wave, which reflect the inner nuclear layer response, were not different between control and ONA animals at all measured light intensities (*p* > 0.05; Figure 2B).

Furthermore, the mRNA expression levels of *Pou4f1* and *Tubb3* (marker for RGCs) were investigated. We could demonstrate a significant downregulation of *Pou4f1* (0.70-fold expression; *p* = 0.036) and *Tubb3* mRNA levels (0.61-fold expression; *p* = 0.006; Figure 1C) in ONA retinae. Additionally, the number of RGCs was elaborated using a specific anti-RBPMS staining (Figure 2D). A significant decrease of RBPMS^+^ RGCs was detected in ONA animals (28.08 ± 1.11 cells/mm) compared to controls (43.90 ± 3.81 cells/mm; *p* = 0.002; Figure 2E).

In addition, RT-qPCR analyses revealed an upregulation of *Gfap* mRNA expression levels in ONA rats (4.83-fold expression; *p* = 0.027; Figure 2F). Astrocytes were further evaluated by staining against anti-GFAP (Figure 2G). The GFAP^+^ staining area revealed no differences between the ONA (3.74 ± 0.57 area [%]/image) and control animals (3.32 ± 0.53 area [%]/image; *p* = 0.594; Figure 2H).

### 3.2. Regulation of HSPs after ONA Immunization

**Figure 3:**
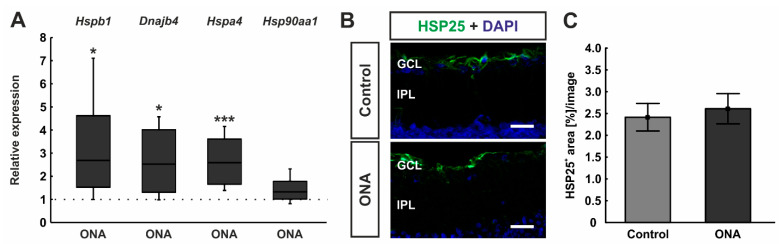
Upregulation of heat shock proteins after immunization. (**A**) The relative expression of *Hspb1* (HSP27) was significantly upregulated in ONA retinae (*p* = 0.036). In addition, the mRNA expression of *Dnajb1* (HSP40; *p* = 0.038) and *Hspa4* (HSP70; *p* < 0.001) were upregulated in ONA retinae compared to controls. The mRNA expression levels of *Hsp90aa1* (HSP90) were not altered after ONA immunization. (**B**) Retinal cross-sections were labelled against anti-HSP25 (green; rat homologue for HSP27), while cell nuclei were counterstained with DAPI (blue). (**C**) The HSP25^+^ area was similar in ONA and control animals. Scale bars: 20 µm. GCL = ganglion cell layer; IPL = inner plexiform layer. Values for RT-qPCR are median± quartile ± minimum/maximum and values for immunofluorescence are mean ± SEM. The dotted line in (**A**) represents the relative expression of the control group. * *p* < 0.05, *** *p* < 0.001.

The mRNA expression levels of different HSPs were examined by RT-qPCR analyses. The relative expression of *Hspb1* (HSP27) was significantly upregulated in ONA retinae (2.69-fold expression; *p* = 0.036). In addition, the mRNA expression of *Dnajb1* (HSP40; 2.53-fold expression; *p* = 0.038) and *Hspa4* (HSP70; 2.59-fold expression; *p* < 0.001) were upregulated in ONA animals compared to controls. *Hsp90aa1* (HSP90) mRNA expression, on the other hand, was not altered after ONA immunization (1.33-fold expression; *p* = 0.102; Figure 3A).

Furthermore, retinae were labelled against anti-HSP25 (rat homologue of HSP27; Figure 3B). The evaluation revealed no alterations in the HSP25^+^ area between ONA (2.61 ± 0.35 area [%]/image) and control animals (2.41 ± 0.32 area [%]/image; *p* = 0.685; Figure 3C).

### 3.3. Involvement of Classical Complement Pathway

**Figure 4:**
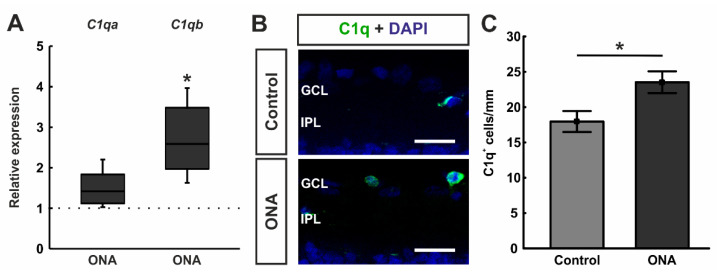
Enhancement of classical pathway components. (**A**) RT-qPCR analyses revealed a trend towards an upregulation in the mRNA expression levels of *C1qa* in ONA retinae (*p* = 0.052), while a significant upregulation of *C1qb* mRNA expression levels was detected in ONA animals (*p* = 0.037). (**B**) A C1q antibody was applied to retinal cross-section (green). DAPI counterstained cell nuclei (blue). (**C**) We noted significantly more C1q^+^ cells in the GCL of ONA animals compared to controls (*p* = 0.023). Scale bars: 20 µm. GCL = ganglion cell layer; IPL = inner plexiform layer. Values for RT-qPCR are median ± quartile ± minimum/maximum and values for immunofluorescence are mean ± SEM. The dotted line in (**A**) represents the relative expression of the control group. * *p* < 0.05.

RT-qPCR analyses of classical pathway components showed a trend towards an upregulation in the mRNA expression levels of *C1qa* in the ONA retinae (1.42-fold expression; *p* = 0.052). A significant upregulation of *C1qb* mRNA expression was noted in ONA rats (2.59-fold expression; *p* = 0.037; Figure 4A).

Cross-sections of ONA and control retinae were stained against anti-C1q (Figure 4B). In accordance with RT-qPCR results, we observed significantly more C1q^+^ cells in the GCL of ONA animals (23.53 ± 1.54 cells/mm) compared to control retinae (17.96 ± 1.48 cells/mm; *p* = 0.023; Figure 4C).

### 3.4. Increased Inflammatory Cytokines

**Figure 5:**
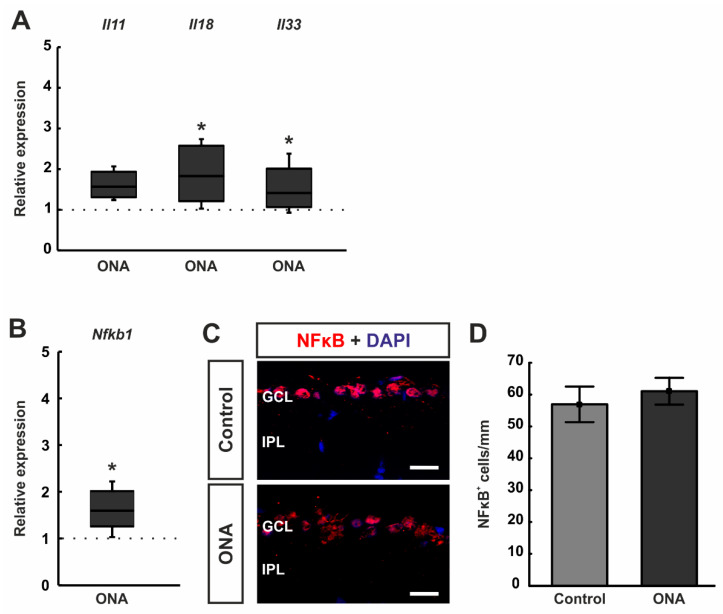
Upregulation of inflammatory cytokines. (**A**) The mRNA expression levels of *Il11* tended towards an upregulation after ONA immunization (*p* = 0.068). A significant upregulation was noted in the mRNA expression levels of *Il18* (*p* = 0.029) and *Il33* (*p* = 0.041) in ONA retinae. (**B**) RT-qPCR analyses of *Nfkb1* mRNA levels revealed a significant upregulation in ONA retinae (*p* = 0.012). (**C**) Retinae were stained against anti-NFκB (green), while DAPI (blue) labelled cell nuclei. (**D**) The number of NFκB^+^ cells were not altered between both groups. Scale bars: 20 µm. GCL = ganglion cell layer; IPL = inner plexiform layer. Values for RT-qPCR are median ± quartile ± minimum/maximum and values for immunofluorescence are mean ± SEM. The dotted lines in (**A**,**B**) represent the relative expression of the control group. * *p* < 0.05.

Different inflammatory cytokines were elaborated in RT-qPCR analyses. The mRNA expression levels of *Il11* tended towards an upregulation after ONA immunization (1.57-fold expression; *p* = 0.068). A significant upregulation was noted in the mRNA expression levels of *Il18* (1.83-fold expression; *p* = 0.029) as well as *Il33* (1.41-fold expression; *p* = 0.041; Figure 5A).

Additionally, RT-qPCR analyses of mRNA levels of the transcription factor *Nfkb1* revealed a significant upregulation in the ONA retinae (1.59-fold expression; *p* = 0.012; Figure 5B).

Furthermore, retinae were stained against the transcription factor NFκB (Figure 5C). Here, the number of NFκB^+^ cells was not altered between ONA (61.07 ± 4.20 cells/mm) and control rats (56.95 ± 5.59 cells/mm; *p* = 0.566; Figure 5D).

### 3.5. Regulated Genes in Microarray Analysis

**Figure 6:**
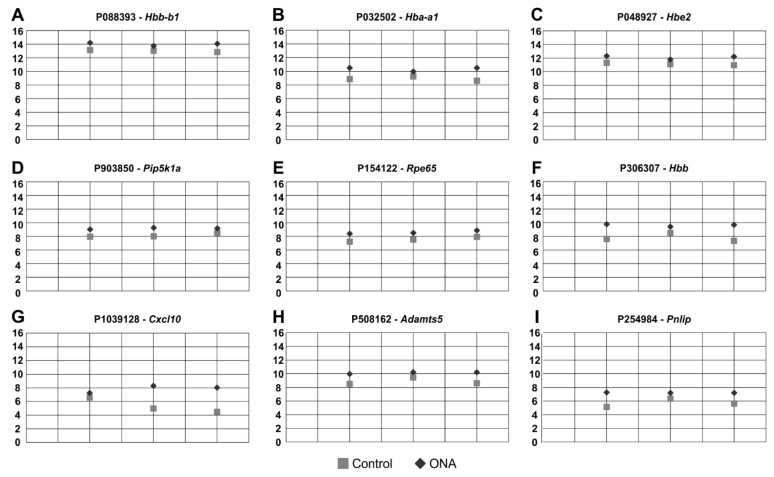
Upregulated oligonucleotide probes. (**A**–**I**) These nine oligonucleotide probes were found upregulated in ONA animals following microarray analysis of the inner retinal layers. All plots display the normalized fluorescence intensity (y-axis) of ONA and control animals (x-axis).

**Figure 7:**
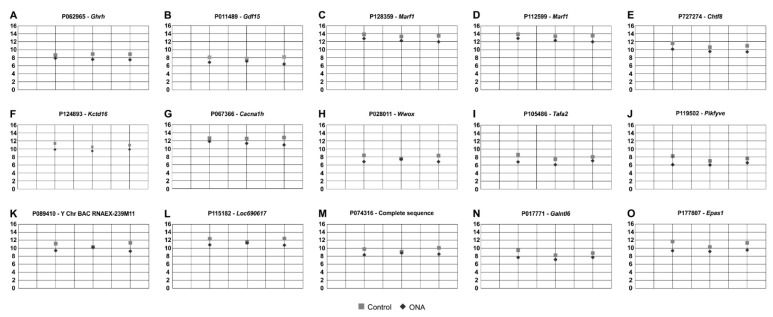
Downregulated regulated oligonucleotide probes. (**A**–**O**) The 15 oligonucleotide probes found downregulated in ONA rats following microarray analysis of the inner retinal layers. All plots display the normalized fluorescence intensity (y-axis) of ONA and control animals (x-axis).

RNA was isolated from the inner layers (GCL, IPL, and INL) of individual retinae from both groups. T-test (*p* < 0.050) revealed 24 significant expression differences between the ONA and control animals. Nine of these probes were upregulated (Table 3; Figure 6) in EAG animals, while fifteen were downregulated (Table 4; Figure 7).

Based on the results of the microarray assays, we performed additional RT-qPCR analyses of the most up- and downregulated genes (*p* < 0.05). Amongst others, *Hba-a1* (*p* = 0.006), *Hbe2* (*p* = 0.009), and *Cxcl10* (*p* = 0.039) were found upregulated in the ONA retinae in the microarray analysis. 

**Figure 8:**
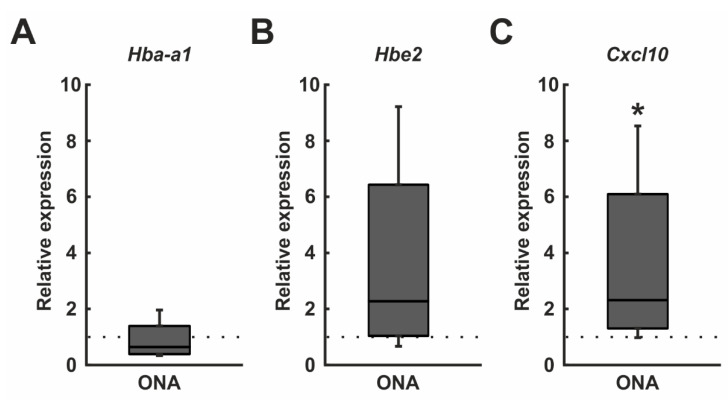
*Cxcl10* upregulation. (**A**) RT-qPCR analyses revealed no differences in the *Hba-a1* mRNA levels in ONA retinae. (**B**) The mRNA expression levels of *Hbe2* were unaltered after ONA immunization. (**C**) An upregulation of the *Cxcl10* mRNA expression levels was found in the ONA group (*p* = 0.033). Values are median ± quartile ± minimum/maximum. The dotted lines represent the relative expression of the control group. * *p* < 0.05.

RT-qPCR analysis revealed no alterations in mRNA expression levels of *Hba-a1* in ONA retinae (0.64-fold expression; *p* = 0.202; Figure 8A). Additionally, *Hbe2* mRNA expression levels were unaltered after ONA immunization (2.28-fold expression; *p* = 0.204; Figure 8B). An upregulation of the mRNA expression levels of *Cxcl10* was found in the ONA group in RT-qPCR experiments (2.33-fold expression; *p* = 0.033; Figure 8C).

As mentioned, in the microarray assays, 15 oligonucleotide probes were downregulated in the ONA retinae, for example *Ghrh* (*p* = 0.002), *Gdf15* (*p* = 0.011), and *Wwox* (*p* = 0.030; Table 4; Figure 7).

**Figure 9:**
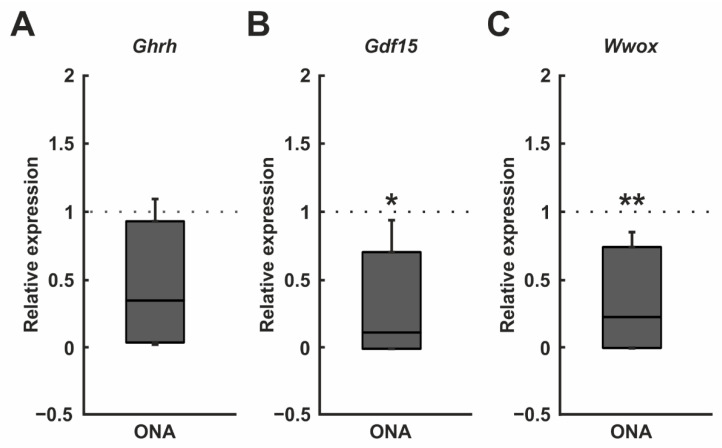
Downregulation of *Gdf15* and *Wwox*. (**A**) RT-qPCR analyses of *Ghrh* mRNA levels revealed no significant differences between both groups. (**B**) The mRNA expression levels of *Gdf15* were downregulated in the ONA samples (*p* = 0.016). (**C**) A downregulation of the *Wwox* mRNA expression was detected in the ONA group (*p* = 0.002). Values are median ± quartile ± minimum/maximum. The dotted lines represent the relative expression of the control group. * *p* < 0.05, ** *p* < 0.01.

The subsequent RT-qPCR analyses of *Ghrh* mRNA levels revealed a trend towards a downregulation in the ONA retinae (0.35-fold expression; *p* = 0.074; Figure 9A). After ONA immunization, the mRNA expression levels of *Gdf15* were significantly decreased (0.12-fold expression; *p* = 0.016; Figure 9B). It was also found that the mRNA expression levels of *Wwox* were downregulated in the ONA group (0.24-fold expression; *p* = 0.002; Figure 9C).

## 4. Discussion

Glaucoma is currently the second most common cause of blindness worldwide and occurs mainly in the elderly [35]. Due to an aging society, there will be an increase of severe visual impairment and blindness in the coming years. By 2040, the projected number of people suffering from glaucoma will rise to about 7.8 million in Europe alone [36]. For this reason, there is an urgent need for developing curative therapies. Currently, only symptomatic, but no disease-modifying treatment options are available. The treatment focus has been on IOP lowering. However, there are also glaucoma forms without elevated IOP, or patients treated with IOP-lowering therapy that still show disease progression. In addition, medical IOP-lowering treatment has serious side effects which leads to poor patient compliance [37]. To identify novel treatment options in the future, a better understanding of underlying pathomechanisms is needed.

As shown previously, ONA immunization in rats leads to a loss of RGCs after 28 days [21,24], which was also observed in the current study. Retinal function, measured with ERG, was not altered in the ONA animals. However, the function of RGCs should be investigated in future studies by using either pattern ERG or the photopic negative response as both techniques are better suited for this purpose. The decline of RGCs in the ONA group in our study was accompanied by a significant upregulation of *Gfap* mRNA levels, while the measured GFAP^+^ area was not altered. Noristani et al. showed significantly more GFAP protein using Western blot, but not by immunohistological staining 28 days after ONA immunization [21]. Moreover, in the transgenic “Connective Tissue Growth Factor” (CTGF) POAG mouse model, alterations in the GFAP expression could only be observed by RT-qPCR [38]. It seems that for analyses of macroglia, the evaluation of antibody staining is not sufficient to detect changes. Nonetheless, the increase in *Gfap* mRNA levels revealed a possible reactive gliosis, which occurs in many retinal diseases [39,40,41].

In the EAG model, a wide range of the immune responses have already been investigated. The study results include an activation of microglia and the complement system [21,25]. Furthermore, HSPs were reported to be involved in glaucomatous neurodegeneration in the EAG model [23,30,42]. HSPs belong to a superfamily of stress proteins. The superfamily can be subdivided into several subfamilies, based on their molecular weight. Primarily, HSPs occur under physiological conditions as chaperones and can have antiapoptotic activities [43,44]. Furthermore, they can stimulate the innate as well as the adaptive immune system [16,17]. In our study, an upregulation of HSP27 (*Hspb1*), HSP40 (*Dnaj4*), and HSP70 (*Hspa4*) was noted in ONA retinae 28 days after immunization. Generally, HSPs are thought to have protective functions, such as stabilization of proteins and the reduction of apoptosis [45]. HSP70 is one of the larger proteins found in the cytosol, endoplasmic reticulum, mitochondria, nucleus, and in the extracellular environment [46]. HSP27, on the other hand, is one of the small HSPs and is ubiquitously expressed and involved in several biological functions [47]. Cytochrome c-mediated activation of caspases in the cytosol can be inhibited by HSP intracellularly [48] and extracellularly and serves as a signaling molecule and binds to membrane receptors [49]. As reviewed by Tsai et al., HSP27 and HSP70 are already strongly connected to glaucoma disease [50]. In patients with normal tension glaucoma (NTG), an enhanced serum immunoreactivity against several antibodies was noted, including HSP27 [51]. Furthermore, in serum samples of POAG and NTG patients, antibodies against HSP27 were detected among others [52]. In rats, immunization with HSP27 ultimately led to glaucomatous neurodegeneration without IOP elevation [23,53]. Additionally, the local administration of HSP27 by intravitreal injections degenerates RGCs and optic nerves [54]. In the case of HSP70, an upregulation was detected in aqueous humor samples of NTG patients [13]. In POAG, higher HSP70 levels were noted in serum specimens [55]. The gained results from our study as well as the current literature underline the pivotal role of HSPs in glaucoma disease. Whether they act neuroprotective or neurodestructive needs to be determined in future studies using specific inhibitors. Studies revealed that HSP70 can act as “chaperokine”, meaning it can act as chaperone as well as cytokine. HSP70 is able to induce the activation of the classical complement pathway independently from antibodies [56]. In the EAG model, an activation of the complement system could be detected previously [25,57]. While prior to RGC loss, the lectin pathway seems to activate the complement cascade, we now revealed that later, the classical pathway is also involved.

In contrast to HSP27 and HSP70, little is known about HSP40 in glaucoma. However, in rheumatic arthritis, oral HSP40 application leads to reduction of disease symptoms, which is most likely due to a reduction of proinflammatory cytokines in addition to an increased production of regulatory cytokines [58].

Hence, it seems likely that there is a connection between the response of HSP70 and the complement system in our IOP-independent glaucoma model. A contribution of the complement response in glaucomatous optic neuropathy was intensively investigated over the last few years. It could be shown that not only in glaucoma animal models with and without IOP elevation [25,59,60,61], but also in human glaucoma [6,19], the complement system triggers degeneration. These results suggest that it could serve as a disease marker in the future. An activation of the complement response can engage in the regulation of an inflammatory reaction [62]. In our study, we noted an upregulation of *Il18* and *Il33* mRNA levels after ONA immunization. Both interleukins belong to the IL1 cytokine family [63]. An increase in IL18 was also observed in an induced ocular hypertension model [64] as well as in high-pressure DBA2/J mice [65], suggesting a role of this interleukin in glaucoma pathogenesis. Unlike other members of the IL1 family, IL33 is constitutively expressed, for example, in glia as well as in some immune cells [66]. For glaucoma, the role of IL33 was not investigated yet. In an experimental uveitis model, IL33 attenuated the development of the disease [67]. In retinal pigment epithelium cells (RPE), IL33 seems to be a key regulator for metabolism and primary RPE cells from IL33^-/-^ mice had significant changes in the structure of mitochondria [68]. Furthermore, IL33 can induce NFκB. This is in accordance with our results since we also found a significant upregulation of *Nfkb1* mRNA levels in ONA retinae.

In this study, we additionally used mRNA profiling to elucidate the relevance of specific markers. In a previous study on photoreceptor degeneration in a mouse model, it was shown that microarray analysis is feasible to perform gene expression analysis [69]. Thereby, they used Cngb1^−^/^−^ mice to investigate the extent of microglia activation in retinal degeneration as well as gene expressions of Cngb1^−^/^−^ and respective wildtype animals. In our study, we were able to detect significant expression differences between the ONA and control animals for the first time. A significant upregulation of 9 of these genes and a downregulation of 15 genes in EAG animals was verified (*p* < 0.050). These results indicate the importance of several genes involved in glaucoma. To further investigate those found changes, we performed RT-qPCR to explore if these changes also occur on the mRNA level of the total retina. We observed an increase in *Cxcl10* mRNA expression level in the ONA group. It is known that Cxcl10 has a role in the recruitment of inflammatory cells and neurodegenerative diseases [70]. In a more recent study, *Cxl10* expression levels were significantly elevated in traumatic optic neuropathy in 8 to 12-week-old mice. Additionally, this demonstrated that Cxcl10 plays a role in leukocyte recruitment and neuronal injury [71]. Additionally, in high-pressure βB1-CTGF mice, an upregulation of *Cxcl10* was noted in the transgenic animals, which was accompanied by an activation of the complement system by the classical pathway [59]. In the study presented here, similar pathways seem to be involved in glaucomatous damage.

Furthermore, we observed a downregulation of the mRNA expression levels of *Gdf15*. *Gdf15* is associated with RGC death [72]. In an in vivo mouse model, the intravitreal injection of GDF15 suppressed RGC loss in the mice and was, therefore, thought to have a protective effect on RGCs [73]. In a study by Ban et al., *Gdf15* expression was specifically increased in the retina after acute RGC axon injury and in a chronic mouse glaucoma model [72]. Gdf15 may be a good marker to screen for glaucomatous neurodegeneration. However, further studies are needed to gain more detailed knowledge about Gdf15. In addition, we observed a downregulation of the mRNA expression level of *Wwox*. Targeted disruption of *Wwox* is known to cause neurodevelopmental abnormalities in mice. These include, among others, abnormal neuronal differentiation, migration of the central nervous system, degenerative alteration, and optic nerve atrophy [74]. In another study, analyses were collected from a family affected by multiple pre- and postnatal abnormalities, including severe neurodevelopmental disorders and refractory epilepsy [75]. A segregating homozygous *Wwox* mutation was detected in this family, resulting in defective architecture of granular and molecular cell layers [75]. The findings of previous studies and our results have in common that loss of *Wwox* can affect and alter various components of the central nervous system, suggesting a regulatory role of *Wwox* in the retina.

## 5. Conclusions

In this study, we noted a significant loss of RGC in ONA retinae. Using DNA microarray for the first time, we detected novel oligonucleotide probes, which might contribute to glaucomatous neuropathy. Furthermore, we observed an upregulation of mRNA expression levels of HSP family genes. Moreover, mRNA expression levels of genes involved in the regulation of the immune system, such as *C1qb*, *Nfkb1*, and *Cxcl10*, were increased in ONA animals. Collectively, these findings provide further insight into the central role of the immune response in glaucomatous optic neuropathy, which could lead to new diagnostic and therapeutic options.

## Figures and Tables

**Table 1:** Sequences of oligonucleotide pairs. The listed primer pairs were used in quantitative real-time PCR experiments, while *Actb* and *Ppid* served as housekeeping genes. The predicted amplicon sizes are given. F = forward, R = reverse, bp = base pair.

Gene	Forward (F) and Reverse (R) Oligonucleotides	GenBank Accession Number	Amplicon Size
*Actb*-F *Actb*-R	cccgcgagtacaaccttct cgtcatccatggcgaact	NM_031144.3	72 bp
*C1qa-*F *C1qa-*R	cgggtctcaaaggagagagag ccagattcccccatgtctc	XM_032888144.1	88 bp
*C1qb*-F *C1qb-*R	gcactccagggataaaagga ccctttctctcctaactcacca	NM_019262.2	74 bp
*Cxcl10*-F *Cxcl10-*R	caagtgctgctgtcgttctc atctcaacatgcggacagga	NM_139089.2	178 bp
*Dnajb1*-F *Dnajb1-*R	attttcgaccgctatggaga cattagcaccaccactgctc	NM_001108441.1	73 bp
*Gdf15-*F *Gdf15*-R	tcagctgaggttcctgctgttc gctcgtccgggttgagttg	NM_019216.2	128 bp
*Gfap*-F *Gfap-*R	tttctccaacctccagatcc gaggtggccttctgacacag	NM_017009.2	64 bp
*Ghrh*-F *Ghrh-*R	tttgtgctcctcaccctcac aattggcccaggattctccg	NM_031577.1	122 bp
*Hba-a1*-F *Hba-a1-*R	ccctggagatttcacacccg aacggtacttggaggtcagc	NM_013096.2	86 bp
*Hbe2*-F *Hbe2-*R	cggccatcatgggtaatccc agaagaaagaacaatcaccagcac	NM_001024805.1	194 bp
*Hsp90aa1-*F *Hsp90aa1-*R	gggagctcatttccaactcc gggttcggtcttgcttgtt	NM_175761.2	129 bp
*Hspa4-*F *Hspa4-*R	catatccaatatctttgaggtgga tggggaagacttcacagtca	NM_153629.1	69 bp
*Hspb1-*F *Hspb1-*R	gaggagctcacagttaagaccaa ttcatcctgcctttcttcg	NM_031970.4	72 bp
*Il11-*F *Il11-*R	gctggtccttccctaaagactc aaggctaggcgagacatcaa	NM_133519.4	104 bp
*Il18-*F *Il18-R*	gcctgatatcgaccgaaca ccttccatccttcacagatagg	NM_019165.1	112 bp
*Il33-*F *Il33-*R	gcaaagtgcgacagcaca ctttggtcttctgttgggatct	NM_001014166.1	76 bp
*Nfkb1-*F *Nfkb1-*R	ctggcagctcttctcaaagc ccaggtcatagagaggctcaa	NM_001276711.1	70 bp
*Pou4f1-*F *Pou4f1-*R	ctggccaacctcaagatcc cgtgagcgactcgaacct	XM_008770931.2	72 bp
*Ppid-*F *Ppid-*R	tgctggaccaaacacaaatg cttcccaaagaccacatgct	M19553.1	88 bp
*Tubb3-*F *Tubb3-*R	tccagctcactcactcactg ggtctcatccgtgttctcca	NM_139254.2	200 bp
*Wwox-*F *Wwox-*R	cagagatacgacgggagcac gctccagtaaccaggaccac	NM_001106188.1	83 bp

**Table 2:** Primary and secondary antibodies used for immunohistology.

Primary Antibodies			Secondary Antibodies		
Antibody	Company	Dilution	Antibody	Company	Dilution
Anti-C1q	Abcam	1:400	Donkey anti-rabbit Alexa Fluor 488	Jackson Immuno Research	1:500
Anti-GFAP	Millipore	1:2000	Donkey anti-chicken Alexa Fluor 488	Jackson Immuno Research	1:500
Anti-HSP25	Enzo Life Science	1:100	Goat anti-rabbit Alexa Fluor 488	Invitrogen	1:500
Anti-NFκB	Santa Cruz	1:300	Donkey anti-mouse Alexa Flour 555	Abcam	1:500
Anti-RBPMS	Millipore	1:500	Donkey anti-rabbit Alexa Fluor 555	Invitrogen	1:500

**Table 3:** Oligonucleotide probes, which were found upregulated in ONA animals.

Probe Name	Gene Symbol	Description	*p*-Value	Fold Change ONA vs. Control
A_64_P088393	Hbb-b1	Rattus norvegicus hemoglobin, beta adult major chain (Hbb-b1), mRNA [NM_198776]	0.006	1.077 (↑)
A_64_P032502	Hba-a1	Rattus norvegicus hemoglobin, alpha 1 (Hba1), mRNA [NM_013096.2]	0.006	1.159 (↑)
A_64_P048927	Hbe2	Rattus norvegicus hemoglobin, epsilon 2 (Hbe2), mRNA [NM_001024805]	0.009	1.088 (↑)
A_44_P903850	Pip5k1a	Rattus norvegicus phosphatidylinositol-4-phosphate 5-kinase, type 1, alpha (Pip5k1a), mRNA [NM_001042621]	0.017	1.123 (↑)
A_64_P154122	Rpe65	Rattus norvegicus RPE65, retinoid isomerohydrolase (Rpe65), mRNA [NM_053562]	0.018	1.136 (↑)
A_44_P306307	Hbb	Rattus norvegicus hemoglobin subunit beta (Hbb), mRNA [NM_033234]	0.027	1.231 (↑)
A_44_P1039128	Cxcl10	Rattus norvegicus C-X-C motif chemokine ligand 10 (Cxcl10), mRNA [NM_139089]	0.039	1.470 (↑)
A_44_P508162	Adamts5	Rattus norvegicus ADAM metallopeptidase with thrombospondin type 1 motif, 5 (Adamts5), mRNA [NM_198761]	0.042	1.144 (↑)
A_44_P254984	Pnlip	Rattus norvegicus pancreatic lipase (Pnlip), mRNA [NM_013161]	0.049	1.264 (↑)

**Table 4:** Oligonucleotide probes, which were found downregulated in ONA animals.

Probe Name	Gene Symbol	Description	*p*-Value	Fold Change ONA vs. Control
A_64_P062965	Ghrh	Rattus norvegicus growth hormone releasing hormone (Ghrh), mRNA [NM_031577]	0.002	0.866 (↓)
A_64_P011489	Gdf15	Rattus norvegicus growth differentiation factor 15 (Gdf15), mRNA [NM_019216]	0.011	0.849 (↓)
A_64_P128359	Marf1	meiosis arrest female protein 1-like [Source:RGD Symbol;Acc:9219913] [ENSRNOT00000003193]	0.019	0.912 (↓)
A_64_P112599	Marf1	Rattus norvegicus meiosis arrest female 1 (Marf1), mRNA [NM_133421]	0.020	0.912 (↓)
A_44_P727274	Chtf8	Rattus norvegicus chromosome transmission fidelity factor 8 (Chtf8), mRNA [NM_001194951]	0.021	0.877 (↓)
A_64_P124893	Kctd16	Rattus norvegicus potassium channel tetramerization domain containing 16 (Kctd16), mRNA [NM_001172155]	0.022	0.890 (↓)
A_64_P067366	Cacna1h	Rattus norvegicus calcium voltage-gated channel subunit alpha1 H (Cacna1h), mRNA [NM_153814]	0.028	0.900 (↓)
A_64_P028011	Wwox	Rattus norvegicus WW domain-containing oxidoreductase (Wwox), mRNA [NM_001106188]	0.030	0.864 (↓)
A_64_P105486	Tafa2	PREDICTED: Rattus rattus TAFA chemokine like family member 2 (Tafa2), transcript variant X2, mRNA [XM_032913314.1]	0.036	0.826 (↓)
A_64_P119502	Pikfyve	phosphoinositide kinase, FYVE-type zinc finger containing [Source:RGD Symbol;Acc:1592067] [ENSRNOT00000020447]	0.040	0.816 (↓)
A_64_P089410	Complete sequence	Rattus norvegicus Y Chr BAC RNAEX-239M11 (Amplicon Express Rat SHR-Akr BAC library) complete sequence [AC244815.7]	0.043	0.881 (↓)
A_64_P115182	LOC690617	Rattus norvegicus hypothetical protein LOC690617 (LOC690617), mRNA [NM_001109605]	0.044	0.909 (↓)
A_64_P074316	Complete sequence	Rattus norvegicus, 25 clones, strain BN/SsNHsdMCW RNOR03324481, whole genome shotgun sequence [AABR03128056]	0.044	0.885 (↓)
A_64_P017771	Galntl6	Rattus norvegicus polypeptide N-acetylgalactosaminyltransferase-like 6 (Galntl6), mRNA [NM_001135756]	0.046	0.851 (↓)
A_44_P177807	Epas	Rattus norvegicus endothelial PAS domain protein 1 (Epas1), mRNA [NM_023090]	0.047	0.844 (↓)

## Data Availability

Not applicable.

## Supplementary Materials

The following supporting information can be downloaded at: https://www.mdpi.com/article/10.3390/biom12101538/s1, Supplement Table S1: Raw data of microarray analysis.
